# Water flow across the interface of contrasting materials: Pressure discontinuity and its implications

**DOI:** 10.1016/j.jhydrol.2018.09.029

**Published:** 2018-11

**Authors:** Zhongyang Li, Di Wang, Xiaoxian Zhang, John W Crawford

**Affiliations:** a Farmland Irrigation Research Institute, Chinese Academy of Agricultural Sciences, Xinxiang 453002, Henan Province, PR China; b School of Engineering, The University of Liverpool, Brownlow Street, Liverpool L69 3GQ, United Kingdom; c Department of Sustainable Agriculture Sciences, Rothamsted Research, West Common, Harpenden, Hertfordshire AL5 2JQ, United Kingdom

**Keywords:** Homogenization, Stratified media, Pore-scale modelling, Pressure discontinuity, Upscaling

## Abstract

Water flow along or across the interfaces of contrasting materials is ubiquitous in hydrology and how to solve them in macroscopic models derived from volumetric average of the pore-scale processes remains elusive. While the change in the average velocity and pressure at water-sediment interface has been well established for channel flow over porous beds, whether a volumetric average alerts the pressure continuity when water flows across the interface of two porous materials is poorly understood despite its imperative implications in hydrological modelling. The primary purpose of this paper is to provide evidences via pore-scale simulations that volumetrically averaging the pore-scale processes indeed yields a discontinuous pressure when water flows across a material interface. We simulated two columns numerically reconstructed by filling them with stratified media: One is an idealised two-layer system and the other one is a 3D column filled by fine glass beads over coarse glass beads with their pore geometry acquired using x-ray computed tomography. The pore-scale simulation is to mimic the column experiment by driving fluid to flow through the void space under an externally imposed pressure gradient. Once fluid flow reaches steady state, its velocity and pressure in all voxels are sampled and they are then spatially averaged over each section perpendicular to the average flow direction. The results show that the average pressure drops abruptly at the material interface no matter which direction the fluid flows. Compared with the effective permeability estimated from the homogenization methods well established in the literature, the emerged discontinuous pressure at the interface reduces the combined ability of the two strata to conduct water. It is also found that under certain circumstances fluid flow is direction-dependant, moving faster when flowing in the fine-coarse direction than in the coarse-to-fine direction under the same pressure gradient. Although significant efforts are needed to incorporate these findings into practical models, we do elicit the emergence of discontinuous pressure at material interface due to volumetric average as well as its consequent implications in modelling of flow in heterogeneous and stratified media.

## Highlights

• Pore-scale model is presented to simulate fluid flow in columns filled with two stratified media.
• Volumetric average of the simulated results yields a pressure discontinuity at the strata interface.
• The emerged discontinuous pressure reduced the combined ability of the media to conduct fluid.
• Under certain circumstance the flow rate in the stratified media is even direction-dependant.

## 1 Introduction

Water flow over or cross the interfaces of different materials is ubiquitous in both surface and subsurface hydrology, and how to solve them is an issue that still attracts interest in modelling of flow in heterogeneous and stratified media (Strack, 2017). Physically, the microscopic water pressure and velocity are continuous and there is no interface between the pore spaces in different materials. In practical models for large scales, however, the delicate pore-scale processes cannot be explicitly resolved and they are instead volumetrically averaged with the impact of the porous structure described by effective parameters, such as permeability for fluid flow and dispersion coefficient for solute transport (Simunek et al., 2003). Material interfaces emerge as a result and need to be treated explicitly when solving for the volumetric average flow rate and pressure. While mass conservation requires the average flow rate across the interfaces to be continuous, there are no physical criteria for the average pressure to meet. Therefore, it has long been speculated that a volumetric average could render what are continuous at pore space discontinuous at macroscopic scales (Berkowitz et al., 2009). For example, it has been found in channel flow over porous bed that the velocity jumps at the water-sediment interface as evidenced from experimental data that, compared to water flow over an impermeable bed, a porous bed could greatly enhance the flow rate (Beavers and Joseph, 1967). Beavers and Joseph (1967) derived a formula to describe this velocity jump, which, known as Beavers-Joseph model in the literate since (Nield, 2016), has been used to simulate flows involving fluid-sediment interfaces such as water flow in karst aquifers (Hu et al., 2012). Early applications of the Beavers-Joseph model assumed a continuous pressure around the interface (Sahraoui and Kaviany, 1992), but recent work has revealed that this might not be true. For example, numerical simulations showed that the pressure at the water-sediment interface is continuous only when the sediment is isotropic and becomes discontinuous if the sediment is anisotropic (Carraro et al., 2013). For water infiltration into a sand bed from channel, it was also found that the average pressure could become discontinuous (Carraro et al., 2015).

The aforementioned efforts were for channel flow over sediment beds with water flow in the sediments described by the Darcy law. For heterogeneous and stratified soils and aquifers, water can move either along or across the interfaces of different materials. How the pressure changes across such interfaces remains elusive and is poorly documented (Nick and Matthai, 2011). A common conjecture in most macroscopic models is that, given that the fluid pressure in void space is continuous, a volumetric average of the pore-scale processes should not alter this continuity (Gohardoust et al., 2017). This is the key assumption used in most homogenization methods, such as the wavelet transformation method (King, 1989, Moslehi et al., 2016), to estimate the effective permeability of heterogeneous and stratified porous formations. For example, it has been well established and routinely used that the effective permeability of a saturated layered system equals to the harmonic mean and arithmetic mean of the individual permeability of each layer for flow parallel and perpendicular to the layers respectively (Mualem, 1984); these were proven even applicable to estimate effective permeability of unsaturated stratified soils if the individual layers are not too thick (Yeh et al., 1985). It is worth pointing out that the above conclusion is valid only if the pressure at the strata interfaces is continuous, which has yet been proven. To the contrary, theoretical analysis of immiscible flow suggested a discontinuous pressure at material interface (Hassanizadeh and Gray, 1989), but evidences proving or disapproving such a discontinuity are lack even for single-phase flow due to the difficulty associated with measuring fluid pressure on each side of a material interface. In the meantime, experimental and theoretical studies on chemical transport in stratified media have both found a mass accumulation when solute moves across material interfaces, suggesting existence of a discontinuous concentration which renders chemical transport in stratified media direction-dependant (Berkowitz et al., 2009, Zhang et al., 2010). Efforts have been made on how to incorporate such discontinuities into macroscopic model for solute transport by assuming the concentration discontinuity is solely caused by permeability difference in the strata (Zoia et al., 2010). This is at odds with some pore-scale simulations which showed that knowing the permeability difference alone is insufficient to quantity the concentration discontinuity and that it is the pore geometry of the adjacent strata that controls how the concentration changes in the proximity of their interface (Zhang et al., 2010).

Given the importance of pressure continuity in modelling fluid flow in heterogeneous and stratified media and the difficulty of experimentally measuring it, we investigated the pressure change across material interface via pore-scale modelling in this paper. We considered single phase flow, and the pore-scale simulations were to mimic column experiment by driving the fluid to flow under an externally imposed pressure gradient. We simulated two columns with each packed by a fine medium and a coarse medium. The first one was an idealised stratified column with a high porosity, and the second one was a 3D column acquired using x-ray computed tomography. In each simulation, when fluid flow was deemed to have reached steady state, we sampled the fluid pressure and the velocity in each voxel and then spatially averaged them cross the sections perpendicular to the average flow direction. Considering that solute transport in two-layer system had been found to be directionally dependant, for each column we also simulated fluid flow in the fine-coarse direction and the coarse-fine direction, respectively, in attempts to examine if fluid flow in the two-layer columns was also direction-dependant.

## 2 Pore-scale simulations

**Fig. 1:**
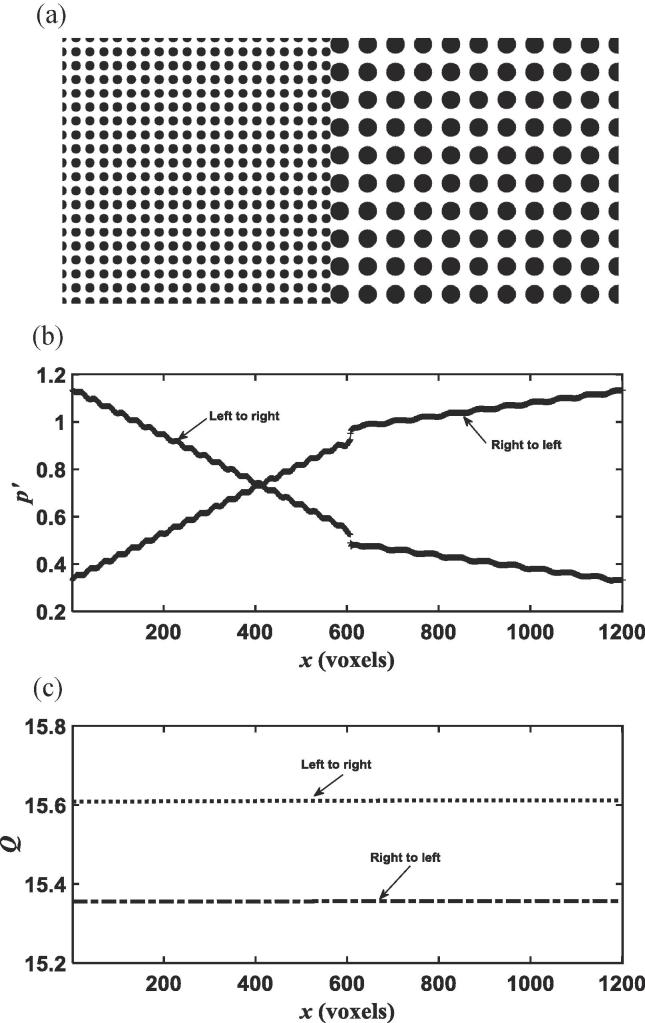
The idealised stratified column for pore-scale simulation (a); the average pressure along the column when fluid flows from the left to the right and from the right to the left respectively (b); average flow rate through the column calculated directly from pore-scale simulations when fluid flows from the left to the right and the right to the left respectively (c).

**Fig. 2:**
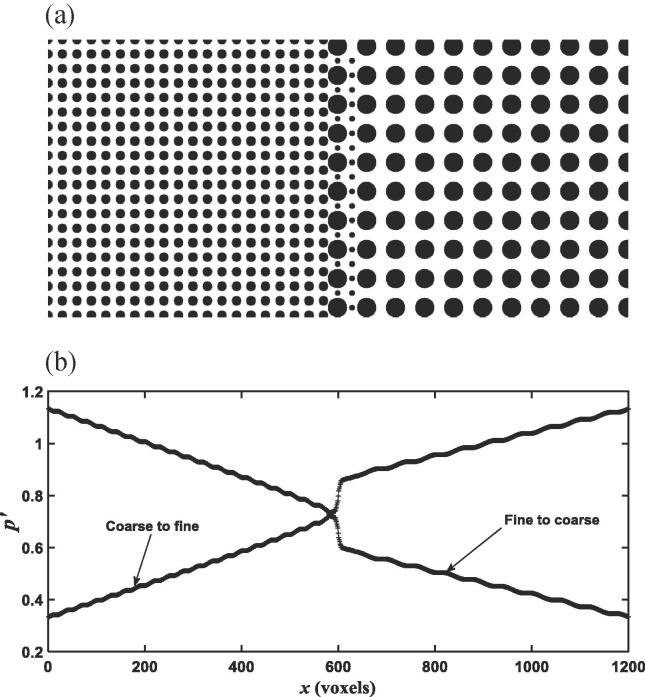
Stratified column with a transitional interface (a); the average pressure along the column when fluid flows in the fine-to-coarse direction and the coarse-to-fine direction (b).

**Fig. 3:**
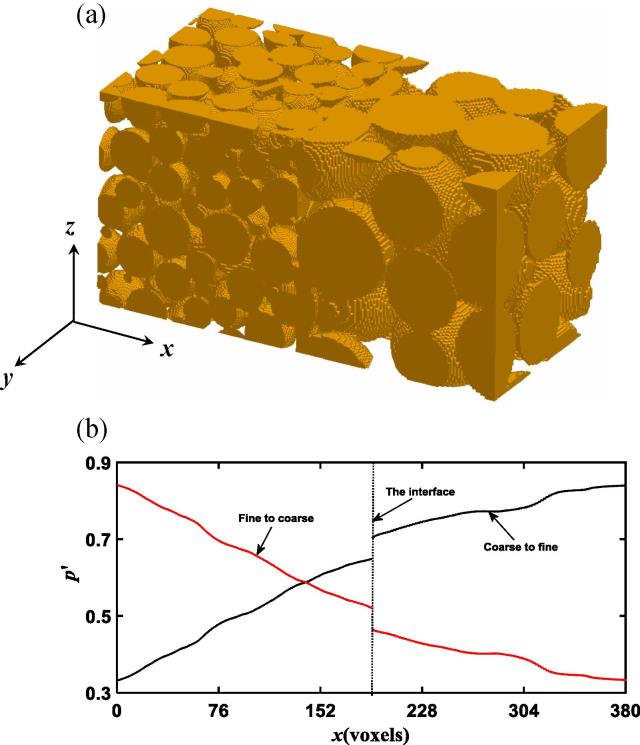
The 3D stratified column acquired using x-ray tomography (a). The average pressure distribution calculated directly from pore-scale simulation when fluid flows in the fine-coarse and the coarse-fine directions respectively (b).

The pore-scale modelling is to test the conjecture that the pressure is continuous at material interfaces after a volumetric average. Fig. 1, Fig. 2, Fig. 3 show the two stratified systems we studied. The first one is an idealised 2D column with high porosity, and the second one is a column filled with fine glass beads and coarse glass beads; the fine glass beads layer was acquired using x-ray tomography and the coarse one was reconstructed numerically by enlarging the size of all fine glass beads and the pores between them two times equally in all directions (Chen et al., 2009, Chen et al., 2008).

The pore-scale simulation is to mimic column experiment by driving fluid to flow under a pressure gradient imposed externally at the two ends of the columns. Fluid flow in the pore geometry is assumed to be laminar and described by the Navier-Stokes equation; it is simulated using the multiple-relaxation time lattice Boltzmann model as follows (d'Humieres et al., 2002):

(1) $$f_{i}\left(x+\delta te_{i},t+\delta t\right)=f_{i}\left(x,t\right)+M^{-1}SM\left[f_{i}^{\mathrm{eq}}\left(x,t\right)-f_{i}\left(x,t\right)\right],$$

where$f_{i}\left(x,t\right)$ is the particle distribution function at location ***x*** and time *t* moving at lattice velocity ***e****_i_*, δ*x* is the size of the voxels in the image, δ*t* is time step, $f_{i}^{\mathrm{eq}}\left(x,t\right)$ is equilibrium distribution function, *M* is a transform matrix and *S* is the collision matrix. The product M*f* in Eq. (1) transforms the particle distribution functions to a moment space in which the collision operation $m=SM\left[f_{i}^{\mathrm{eq}}\left(x,t\right)-f_{i}\left(x,t\right)\right]$ is performed. The post-collision result in the moment space is then transformed back to particle distribution functions by *M*^−1^*m*. We use the D3Q19 lattice model in this paper where the particle distribution functions move in 19 directions with 19 velocities: $\left(0,0,0\right)$, $\left(\pm \delta x/\delta t,\pm \delta x/\delta t,0\right)$, $\left(0,\pm \delta x/\delta t,\pm \delta x/\delta t\right)$, $\left(\pm \delta x/\delta t,0,\pm \delta x/\delta t\right)$ and $\left(\pm \delta x/\delta t,\pm \delta x/\delta t,\pm \delta x/\delta t\right)$ (Qian et al., 1992). The collision matrix is diagonal and the terms in it are given as follows:

(2) $$\begin{array}{c} S={\left(s_{0},s_{1},s_{2},s_{3},s_{4},s_{5},s_{6},s_{7},s_{8},s_{9},s_{10},s_{11},s_{12},s_{13},s_{14},s_{15},s_{16},s_{17},s_{18}\right)}^{T},\\ s_{0}=s_{3}=s_{5}=s_{7}=0,\\ s_{1}=s_{2}=s_{9-15}=1/\tau ,\\ s_{4}=s_{6}=s_{8}=s_{16-18}=8\left(2-\tau^{-1}\right)/\left(8-\tau^{-1}\right), \end{array}$$

The fluid simulated by the above model has a kinematic viscosity$\mu =\delta x^{2}\left(\tau -0.5\right)/6\delta t$ and pressure $p=\rho \delta x^{2}/3\delta t^{2}$. The equilibrium distribution functions are defined as follows:

(3) $$\begin{array}{c} f_{i}^{\mathrm{eq}}=w_{i}\left[\rho +\rho_{0}\left(\frac{3e_{i}\cdot u}{c^{2}}+\frac{9{\left(e_{i}\cdot u\right)}^{2}}{2c^{4}}-\frac{3u\cdot u}{2c^{2}}\right)\right],\\ w_{0}=1/3,\\ w_{i}=1/18,\;∥e_{i}∥=\delta x/\delta t\\ w_{i}=1/36\;∥e_{i}∥=\sqrt{2}\delta x/\delta t \end{array}$$

where c=δx/δt and $\rho_{0}$ is a reference fluid density to ensure that the fluid is incompressible when the flow is in steady state(Zou et al., 1995). The bulk fluid density ρ and velocity ***u*** are updated after each time step by

(4) $$\begin{array}{c} \rho =\sum_{i=0}^{18}f_{i},\\ \rho_{0}u=\sum_{i=1}^{18}f_{i}e_{i} \end{array}$$

Implementation of the above model consists of two steps to advance one time step. The first one is to calculate the collision in the moment space and then transform the results back to particle distribution functions, i.e., to calculate $f_{i}^{*}=f_{i}\left(x,t\right)+M^{-1}SM\left[f_{i}^{\mathrm{eq}}\left(x,t\right)-f_{i}\left(x,t\right)\right]$; and the second step is to move the post-collision particular distribution function $f_{i}^{*}$ to position at $x+\delta te_{i}$ in the time period of δ*t*. During the streaming step, whenever $f_{i}^{*}$ hits a solid voxel, it is bounced back to where it was before the streaming to give a non-slip boundary where the bulk fluid velocity is zero. In each simulation, once flow is deemed to have reached steady state, we sample fluid pressure and velocity at each voxel and then average them across each y-z section as shown in Fig. 3a as follows:

(5) $$\begin{array}{c} P\left(x\right)=\frac{\sum_{i=1}^{N_{\mathrm{xy}}}p\left(x,y_{i},z_{i}\right)}{N_{\mathrm{xy}}},\\ q\left(x\right)=\sum_{i=1}^{N_{\mathrm{xy}}}u_{x}\left(x,y_{i},z_{i}\right), \end{array}$$

where *N_yz_* is the number of fluid voxels in the y-z section located at *x*, $p(x,y_{i},z_{i})$ and $u_{x}\left(x,y_{i},z_{i}\right)$ is the pressure and velocity component at voxel located at (*x*, *y_i_*, *z_i_*), respectively. We also calculate the effective permeability of the column based on the simulated velocity field from

(6) $$k=\frac{\mu }{\mathrm{Ng}}\sum_{i=1}^{N}u_{x}\left(x_{i},y_{i},z_{i}\right),$$

where *k* is the effective permeability; *N* is the number of voxels, including all solid and void voxels; $u_{x}\left(x_{i},y_{i},z_{i}\right)$ is the velocity component in the voxel centred at (*x_i_*, *y_i_*, *z_i_*) and *g* is the externally imposed pressure gradient along the column. In addition to the effective permeability of the stratified media, we also calculate the permeability of the fine and the coarse medium separately within each column shown in Fig. 1, Fig. 2, Fig. 3.

## 3 Result analysis

**Fig. 4:**
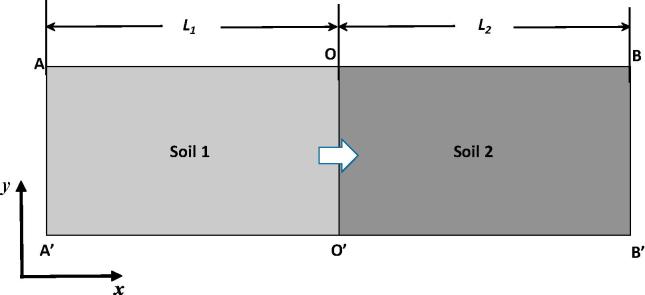
Schematic illustration of the one-dimensional macroscopic flow resulted from volumetric average of the three columns in Fig. 1, Fig. 2, Fig. 3.

After the above volumetric average, the pore-scale flow process in each of the columns is simplified as a one-dimensional macroscopic flow as illustrated in Fig. 4. The two-layer system can be further homogenized using an effective permeability to describe their combined ability to conduct water. If the hydraulic conductivity of Soil 1 and Soil 2 is *k*_1_ and *k*_2_ and their thickness is *L*_1_ and *L*_2_ respectively, the effective hydraulic conductivity *k* of the two soils can be estimated as follows if the pressure at their interface is continuous (Mualem, 1984):

(7) $$\frac{L_{1}+L_{2}}{k}=\frac{L_{1}}{k_{1}}+\frac{L_{2}}{k_{2}}.$$

For the two examples studied in this work $L_{1}=L_{2}$, and the effective hydraulic conductivity is hence $k=2k_{1}k_{2}/\left(k_{1}+k_{2}\right)$. We will call the permeability calculated from Eq. (7) theoretical permeability and compare it with those calculated directly from the pore-scale simulations.

For ease of analysing the simulated results in what follows, the space will be normalized to x′=x/δx, time to $t\prime =t\mu /\delta x^{2}$, density to $\rho \prime =\rho /\rho_{w}$ and pressure to $P\prime =P\delta t^{2}/\delta x^{2}\rho_{w}$, where ρ_w_ is the density of liquid water.

### 3.1 The idealised 2D column

Figure 1b shows the average pressure distributions along the column for fluid flow in the fine-coarse direction and the coarse-fine direction, respectively. It is evident that the pressure is not continuous but endures an abrupt drop at the interface no matter which direction the fluid flowed. Except at the interface, the pressure is continuous and approximately linearly distributed within each of the two strata in the column. Fig. 1c plots the average flow rate along the column calculated from the pore-scale simulations when the fluid flowed in the two opposite directions. The figure shows that under the same pressure gradient, the flow rate is higher when the fluid flowed in the fine-coarse direction than in the coarse-fine direction.

Due to the energy loss and pressure drop across the interface, the real effective permeability of the two soils calculated from pore-scale simulations is smaller than estimated from Eq. (7). Table 1 compares the results. Emergence of the discontinuous pressure at the interface reduced the effective permeability to 8.49 when the fluid flowed in the coarse-fine direction and to 8.63 when it flowed in the fine-coarse direction, compared to the theoretical 9.08 when the pressure is assumed to be continuous.

**Table 1:** Comparison of the effective permeability calculated directly from pore-scale simulations with the theoretical estimates by assuming the pressure at the interface is continuous for the columns shown in Fig. 1, Fig. 3.

	2D Column	3D Column
Permeability of the fine medium *k*_1_	5.89	1.050
Permeability of the coarse medium *k*_2_	19.85	2.452
Theoretical effective permeability	9.08	1.470
Calculated effective permeability in fine-coarse direction	8.63	1.289
Calculated effective permeability in coarse-fine direction	8.49	1.285

The above example is for stratified media with a sharp-cut interface. Stratified geological formations formed naturally usually have transition interfaces where the coarse medium in the proximity of the interface might contain some small-size particles. To elucidate how pressure changes in stratified media with such interfaces, we simulated fluid flow in an idealized image shown in Fig. 2a. The average pressure distribution calculated along the column is shown in Fig. 2b. Strictly speaking, the pressure is more continuous compared to the example shown in Fig. 1a, but it still endured a sharp change and such change cannot be described by Eq. (7) that assumes the pressure is continuous.

### 3.2 The 3D column

The porosity of both the fine and the coarse strata in the 3D column is approximately 37%, much less than the porosity of the 2D idealised column. Fig. 3b shows the average pressure distribution along the column when the fluid flowed in the fine-coarse and the coarse-fine directions. Compared to the 2D columns, the pressure drop across the interface in the 3D column is more significant no matter which direction the fluid flowed, probably because the 3D image is less porous and the energy loss (thus the pressure drop) associated with the flow through it is more significant than that in the 2D idealised example. The key result in this example is that the pressure drop is approximately the same, regardless of flow direction. The example shown in Fig. 3b is for flow under pressure gradient of 0.0013, and the pressure drop over the interface is 0.056. Again, because the energy loss over the interface is almost the same when fluid flow in different directions, their associated permeability is also comparable as shown in Table 1. Strictly speaking, however, the permeability calculated from the pore-scale simulation for flow in the fine-coarse direction is still higher than that in the coarse-fine direction, consistent with the results obtained from the 2D column.

**Fig. 5:**
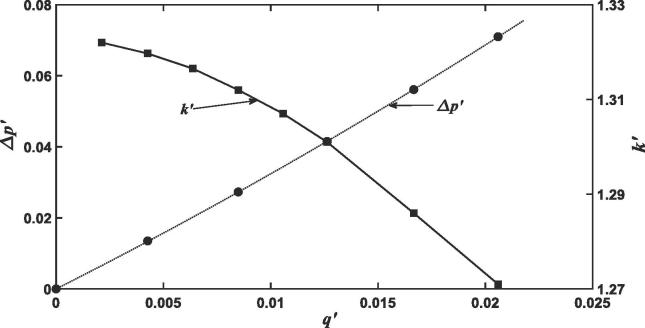
Change in the effective permeability and the pressure drop over the media interface in the 3D column shown in Fig. 4a as the flow rate through it increases.

Physically, the average macroscopic pressure at the strata interface should be continuous when fluid is stagnant, and the pressure drop at the interface is hence solely caused by fluid flow. It is therefore natural to examine how the pressure drop responds to flow rate. Fig. 5 shows the change in the pressure drop as the average flow rate increases. The pressure drop Δ*p* increases parabolically with the average flow rate *q*. Because of the pressure drop and energy loss over the interface, the permeability calculated from the pore-scale simulations decreases as the average flow rate increases as shown in Fig. 5.

## 4 Discussion and conclusions

Pore-scale simulations of water flow in idealised 2D columns and a 3D column obtained using x-ray tomography both revealed that volumetrically averaging the pore-scale process resulted in a macroscopic pressure that is discontinuous at the material interface in the columns. The emerged discontinuous pressure means extra energy loss and, as a result, reduces the combined ability of the two strata to conduct water compared to the prediction from the classical homogenization methods that assume a continuous pressure at the material interface. The magnitude of the pressure drop across the interface varies with physical properties of the materials as well as water flow rate across the column. For the columns we simulated, the pressure drop increases parabolically with water flow rate. Furthermore, depending on physical properties of the strata, water flow could even become direction-dependant in that water moved faster when flowing the fine-coarse direction than in the coarse-fine direction. We also found that a sharp pressure drop existed even for transitional interface in which the coarse medium near the interface contains some small particles.

Early study on channel flow over sediment bed has shown that the change in macroscopic pressure across the water-sediment interface depended on the sediment, being continuous if the sediment is isotropic and discontinuous if the sediment was anisotropic (Carraro et al., 2013, Marciniak-Czochra and Mikelic, 2012). Our simulations suggested that this conclusion appear to be valid only for channel flow in parallel with sand bed and break down when water flows across the interface of two porous materials. For water flow across material interface, the mass conservation requires that the average flow rate calculated from Eq. (5) must be a constant along the column. Physically, the pressure drop at the interface is the consequence of energy loss caused by viscous friction, which increases with velocity. The viscous friction depends on the water-wall interfacial areas, which differ in the fine and coarse media because the specific surface area in the former is bigger than that in the latter. For the 3D column, the porosity of the coarse and the fine medium shown in Fig. 3a is approximately the same, and the average-pore velocity in them is hence also the same. As such, under the same externally imposed pressure gradient, the pressure drop in the 3D column is independent of flow direction as shown in Fig. 3b. In contrast, the porosity of the two media in Fig. 1, Fig. 2 differs slightly and, consequently, the average pore-water velocity in them is different. Therefore, apart from energy loss caused by viscous friction, inertial dissipation due to the abrupt increase or decrease in pore-water velocity might also play a role in inducing the pressure drop. Theoretically, the relative significance of the energy loss caused by viscous friction and inertial dissipation depends on flow rate. However, since water flow in porous materials is viscous, in all columns we simulated, the energy loss is dominated by viscous friction and the pressure drop is hence independent or only slightly dependant of flow direction as evidenced from the simulated results.

Fluid flow in the proximity of material interfaces is ubiquitous in hydrology but complicated to be described. The results presented in this paper might improve our understanding of water flow in heterogeneous and stratified media, but incorporating them into macroscopic models needs substantial efforts even though numerical models capable of dealing with discontinuous pressure at material interfaces exist (Nick and Matthai, 2011). The challenge lies in that the pressure drop across the interface depends not only on material properties and flow rate but also on the flow direction. Quantifying these processes and then incorporating them into macroscopic models is not trivial, especially when flow is transient (Kitanidis, 1990). Given these challenges, assuming a continuous pressure at the material interface is postulated to be the dominant approach in the foreseeable future for modelling flow in heterogeneous and stratified media because of its simplicity in implementation, especially for unsaturated flow which is far more complicated than saturated flow even under steady flow condition (Pruess, 2004). Notwithstanding these, this work still has an important implication as it provides evidence that spatial average (or upscaling) does result in a discontinuous pressure at material interfaces and that the commonly used homogenization methods for estimating effective permeability and for calculating flow across material interfaces in heterogeneous and stratified porous formations could give rise to errors. The significance of the errors depend on media property and flow rate and direction.
